# Reliability of ultrasound to measure morphology of the toe flexor muscles

**DOI:** 10.1186/1757-1146-5-S1-O38

**Published:** 2012-04-10

**Authors:** Karen J Mickle, Christopher J Nester, Gillian Crofts, Julie R Steele

**Affiliations:** 1 Centre for Health Sciences Research, University of Salford, Salford, Greater Manchester, M6 6PU, United Kingdom; 2 Biomechanics Research Laboratory, University of Wollongong, Wollongong, NSW, 2522, Australia

## Background

Measuring the strength of individual foot muscles is very challenging; however, measuring muscle morphology has been shown to be associated with strength [1]. A reliable method of assessing foot muscle atrophy and hypertrophy would therefore be beneficial to researchers and clinicians. Real-time ultrasound (US) is a non-invasive, objective and inexpensive method of assessing muscle morphology and has been employed widely to quantify cross-sectional area (CSA) and linear dimensions of larger muscles (e.g. quadriceps, triceps surae). Few studies, however, have determined its ability to measure the small muscles of the foot and ankle. This study aimed to determine whether US is a reliable tool to measure the morphology of the toe flexor muscles.

## Materials and method

The abductor hallucis (ABH), flexor hallucis brevis (FHB), flexor digitorum brevis (FDB), quadratus plantae (QP) and abductor digiti minimi (ABDM) muscles in the foot and the flexor digitorum longus (FDL) and flexor hallucis longus (FHL) muscles in the shank were assessed in five males and three females (mean age =33.1±11.2 years). Muscles were imaged using a GE Venue 40 US with either a 6-9 or 7.6-10.7 MHz probe in a random order, and on two occasions 1-6 days apart. Muscle thickness and CSA were measured using Image J software with the assessor blinded to muscle and day of scan. Intraclass correlation coefficients (ICC) and limits of agreement (LoA) were calculated to assess day-to-day repeatability of the measurements (Table 1).

**Table 1:** Mean muscle size values taken on Days 1 and 2 and their respective ICC and Limits of Agreement (LoA) values.

	Mean Day 1 (cm)	Mean Day 2 (cm)	ICC	LoA (cm)
ABH CSA	2.45	2.46	0.98	0.45
ABH thickness	1.25	1.20	0.98	0.26
FHB CSA	2.55	2.55	0.83	0.74
FHB thickness	1.36	1.29	0.94	0.22
FDB CSA	2.22	2.32	0.99	0.18
FDB thickness	1.06	1.08	0.87	0.20
QP CSA	1.92	1.91	0.99	0.17
QP thickness	1.09	1.07	0.95	0.20
ABDM CSA	2.28	2.26	0.97	0.37
ABDM thickness	1.14	1.10	0.96	0.20
FDL CSA	1.69	1.57	0.99	0.17
FHL	3.58	4.04	0.98	0.51

## Results and conclusion

The method was found to have good reliability (ICC=0.83-0.99) with LoA between 7.9-29.1% of the relative muscle size. Although published data is not available for all muscles tested, the LoA were within the ranges that we may expect for changes in muscle size due to ageing, disease or intervention. Ultrasound is therefore deemed a reliable method to measure morphology of the toe flexor muscles.
